# Results and Complications of 1104 Surgeries for Velopharyngeal Insufficiency

**DOI:** 10.5402/2012/181202

**Published:** 2012-04-11

**Authors:** Jenő Hirschberg

**Affiliations:** St. John's Hospital, Division of Pediatric Otorhinolaryngology, 1125 Budapest, Diós árok 1-3, Hungary

## Abstract

Velopharyngeal insufficiency (VPI) means that the velopharyngeal closure is inadequate or disturbed. VPI may be organic or functional, congenital or acquired and is caused by structural alterations or paresis. The symptoms are primarily to be found in speech (hypernasality), more rarely in swallowing and hearing. The management types are as follows: speech therapy, surgery, speech bulb, and others. Surgery is indicated if the symptoms of VPI cannot be improved by speech therapy. Among the operative methods, velopharyngoplasty constitutes the basis of the surgery. The pharyngeal flap was incorporated and survived in 98.1% of the cases, hyperrhinophony disappeared or became minimal in 90% after surgery in our material (1104 cases). The speech results seemed to be the same with superiorly or inferiorly based pharyngeal flap. The Furlow technique, push-back procedure, the sphincteroplasty, and the augmentation were indicated by us if the VP gap was less than 7 mm; these methods may also be used as secondary operation. We observed among 1104 various surgeries severe hemorrhage in 5 cases, aspiration in 2 cases, significant nasal obstruction in 68 patients, OSAS in 5 cases; tracheotomy was necessary in 2 cases. Although the complication rate is rare, it must always be considered that this is not a life-saving but a speech-correcting operation. A tailor-made superiorly based pharyngeal flap is suggested today, possibly in the age of 5 years.

## 1. Introduction

Velopharyngeal insufficiency (VPI) means that for whatever reason the performance of the velopharyngeal (VP) closure is inadequate or disturbed. VPI may be organic or functional, congenital or acquired and is caused by structural alterations or paresis. The symptoms and signs of palatal dysfunction are primarily to be found in speech, more rarely in swallowing and hearing. These symptoms depend mainly on the extent of the defect in the closure mechanism, and on the time and manner of its development. If the VPI is consequence of a connatal anomaly or of a perinatal lesion, the first symptoms manifest itself immediately after birth: difficulty or unability of sucking, pharyngeal secret stagnation, vomiting through the mouth and nose, frequent aspirations, attacks of suffocation, repeated pneumonias, and cardiorespiratoire decompensation. The speech disorders are as follows: delayed speech development, hypernasality, nasal escape, articulation disorders (mostly secondary), facial grimaces, and hyperfunctional voice disorders (possibly with vocal nodules). The resonance problem, the hypernasality is among these the leading problem which is an acoustic sign, while the nasal escape is an aerodynamic symptom. In the diagnosis, first routine examinations should be carried out: careful history, intraoral inspection, simple functional tests (Czermak's mirror test, Gutzmann's A-I probe, phonendoscope test), orientative hearing examination, judging of the child's behaviour and intelligibility, and quick teachability test. The instrumental examinations serve for confirming or rejecting the routine procedures; they may be used to define (1) the VP port, its dynamics, the fact, the degree and the type of VP dysfunction (X-ray methods, CT, MRI, endoscopy, ultrasonics), (2) pathological airflow (aerodynamic procedures), (3) speech and voice production (articulation tests, speech intelligibility test, nasometry, acoustic analysis), (4) tube and hearing impairment (audiometry, tympanometry, stapedius reflex test, OAE, BERA), (5) etiological factors (neurological, electrophysiological methods, EMG, biopsy), (6) to judge the personality, intelligence, and stimulability of the patient (IQ test, personality tests and other procedures). In the perceptual evaluation, the use of a four-point rating scale is proposed for the description of all attributes assessed. The treatment of VPI depends on the etiology. Only when it has been unequivocally determined that no pathological process is in the background of VPI, is there justification, scope, and indication for speech (logopedic), phoniatric, or phonosurgical therapy. The following types of treatment may be used: (1) speech therapy, (2) surgery, (3) speech bulb, (4) other treatment (medicaments, electrotherapy). In considering the indication for velopharyngeal surgeries, we must always remember that they are not lifesaving but speech correcting interventions and that surgical complications—though not too often—may also develop. The purpose, the subject of the present article is to evaluate the operative results, and the complications, the sequelae of various surgeries for VPI.

## 2. Material and Methods

The basis of the recent study is about 6000 various facial cleft operations including 1104 velopharyngeal surgeries for VPI performed during 1965–2002 at the pediatric otorhinolaryngological departments of the Heim Pál and Madarász Children's Hospital, Budapest/Hungary.

63.6% of the surgical interventions for VPI were performed as secondary surgery, after staphylorraphy, in cleft palate patients, the rest underwent primary operation indicated in other forms of VPI (Table 1).  Table 2 demonstrates the distribution of the patients according to age: most of them were children aged between 4 and 7 years. The type of the used surgical technique is detailed in Table 3.

The VP closure mechanism, the anatomy of the VP port, the definition, incidence, classification, etiology, genetics, symptomatology, diagnosis, treatment of VPI, and the surgical methods were summarized in my main report of the XXth Congress of the International Association of Logopedics and Phoniatrics, and in Hungarian books [1–3].

The Cleft Palate Committee of IALP (presided over at that time by the author) was regarded as the most informative instrumental diagnostic procedure in diagnosing VPI: the cine (video) fluoroscopy, the nasopharyngoscopy, and the nasometry, supplemented in some cases with electrophysiological methods [4]. This standpoint is accepted since then in our studies.

The Cleft Palate Committee of IALP organized a congress in Visegrád/Hungary about Cleft Palate and Velopharyngeal Insufficiency, where the authors with several specialities (phoniatrics, speech pathology, otorhinolaryngology, audiology, maxillofacial, plastic and pediatric surgery, orthodontics, radiology, pediatrics, and genetics) from all over the world gave a broad survey about many various questions on the topic [5].

A book about the diagnosis and therapy of hypernasality in cases of velopharyngeal insufficiency with and without palatal cleft was published by us in 2006 [6].

In order to define the functional result (speech improvement) and the factors which influence the surgical effect in an approximately objective way, we performed a statistical analysis using *X* ^2^ test on a randomly selected group of 261 patients at least 3 years after surgery with respect to nasality, articulation, and speech intelligibility. First, 3 experienced speech therapists assessed the speech (articulation, nasality) using a four-point rating scale [7], then we applied our speech intelligibility test [8] considering the following factors: age of patient at the time of operation, his hearing and his level of intelligence, VPI etiology, type of surgery, status of the velopharyngeal area after surgery, and postoperative speech therapy.

The surgical complications of our 1104 cases were detected by oral inspection and—if necessary—by nasopharyngoscopy, radiological methods, or the help with repeated intubation.

In judging the operative results, the possible complications, and in indication of a velopharyngoplasty, nasometry may help us. We have used the nasometry adapted to Hungarian language [9] in 160 children to corroborate the listener's detection of hypernasality and in deciding of a velopharyngeal surgery or reoperation in dubious cases.

To decide whether pharyngeal flap surgery has an adverse effect on the growth process of the craniofacial complex (or not), lateral X-ray cephalometry was performed in randomly selected 53 unilateral cleft lip and palate (UCLP) patients with pharyngeal flap surgery: six angular and four linear values were measured on the cephalograms [10]. The control group consisted of 38 UCLP patients without pharyngeal flap surgery. The significance test of the average values was done with the two-sample *t* test. The level of significance was *α* = 5%.

We have used polysomnography [11] among 68 patients still snoring 1 year after surgery for detecting possible obstructive sleep apnea.

## 3. Results

### 3.1. Surgical Results

Surgical results may be judged from the angle of anatomical healing and functional effect.

The anatomical results are good: the pharyngeal flap built in and survived long lasting in 98.1% of the cases. In 94.1% the adhering was complete, in 4% the healing was partial with dehiscences, in 1.9% the flap detached. In cases of augmentation, the implanted material (Teflon, later autologous fat) had never dropped out or had been absorbed and we do not have any observed migration of the implant or significant infection.

Regarding the speech improvement, we have obtained the following functional results: hyperrinophony disappeared or became minimal in 90% and moderate in 10%; speech intelligibility proved to be excellent or good in 74%, acceptable in 21% and poor in 5%. Articulation was excellent in 42% after surgery, the rest of the patients (1–6 sounds defective) were referred for further speech therapy.

The speech improvement depends—at a significance level of *α* = 5%—on the age of the patient (in younger children the results are better than in older children or adults), his hearing and mental level, the etiology of VPI (in cases of structural changes of the soft palate the functional effect is more advantageous than in cases of paresis), and the postoperative anatomical status. According to our survey, the type of surgery (inferiorly versus superiorly based method) and the duration of the postoperative logopedic treatment did not influence the functional effect. The surgeon's experience and the competence of the speech therapist are, of course, important but not measurable factors.

### 3.2. Surgical Complications

In our patient material (1104 cases) surgical complications were rare.

We have observed complications or undesirable sequelae after surgery as follows:

- death: 0 case,
- serious postoperative bleeding: 5 cases,
- transfusion necessary: 5 cases,
- aspiration, tracheotomy: 2 cases,
- detachment of the flap: 21 cases,
- nasal obstruction (snoring 1 year after surgery): 68 patients,
- OSAS (verified by polysomnography): 5 patients,
- widening of the lateral orificii beside the flap because of significant nasal obstruction or OSAS: 11 cases,
- maxillofacial development. In sagittal relation, the maxilla and mandible became more retrognathic in each patients after flap surgery (the anterior facial height does not show any differences); but the observed changes were not significant.

## 4. Discussion and Conclusions

Ever since Rosenthal [12] and then Sanvanero-Rosselli [13] renewed Passavant's [14] and Schönborn's [15] old surgical concept, the question of speech-improving operation has become one of the central subjects of the practice and literature concerned with CP surgery and VPI [16, 17]. Three important questions should be considered: in which cases is surgery indicated, at what age of the patient should surgical intervention be performed, and what types of surgery or procedures are available, of which the best result can be expected with the minimum of risk and complications.

 Creating the structural elements necessary for velopharyngeal closure is the essential goal of surgical correction of velopharyngeal insufficiency [18]. *We recommend surgery *in those cases of organic VPI which imply a persistent condition (i.e., where a progressive neurological process can be precluded), where no results can be expected on the basis of teachability tests or other data of examination or have been achieved by means of speech therapy of 4–6 months. According to an extensive survey by Schneider and Shprintzen [19], 80% of VPI patients receive speech therapy. If this conservative treatment is unsuccessful, or it seems to be hopeless from the outset, surgery should be indicated. As a first step, however, neurological processes should be excluded.

The velopharyngeal surgery is a speech-correcting operation, although sometimes it may improve also the faulty swallowing and conductive hearing problem. Its aim is first of all improving the phonation, the timbre of the voice. *Thus, this surgical intervention belongs (also) to the field of phonosurgery *[20, 21]. Phonosurgery is still an integrative part of phoniatrics. The surgeon's proper place to some discipline is not decisive, it is important, however, that he/she

- should well know not only the anatomy but also the functions of the VP sphincter,
- should perform such kind of operations regularly to have enough experience, and
- should ensure the contribution of the representatives of all disciplines (otorhinolaryngology, speech pathology, audiology, phoniatrics, neurology, oral surgery, genetics, orthodontics, pediatrics) who may take part in the diagnosis and care of patients with VPI.

Most of these surgical interventions are performed secondarily, after palatoplasty, in cleft patients. According to a large survey, secondary VP surgery was performed in 0–40% after various cleft palate operations in different CP centers [6]. In our own material, speech-correcting operation was necessary in 16.8% among 613 CP patients after Langenbeck's staplylorraphy [20]. Further pathologies which may need palatopharyngeal surgery primarily or not too often secondarily, are submucous cleft palate, occult submucous cleft palate, congenital or acquired shortening of the velum, various syndromes (velocardiofacial syndrome = VCFS, Robin sequence), and paresis of the palate.

*Correct judgement and comparison of the functional results*, the speech improvement is difficult: patients concerning age are not unique in the different studies. The indication is inhomogene, criteria and methods of evaluation are different, the data of the subjective and objective evaluation do not correlate.

As the number of the successful surgeries is given in various publications to be between 67% and 97%, in general the functional results are published to be about 80–90%.

There are several factors which may influence the functional results according to many publications: age of the patient at the time of operation, etiology of the VPI, hearing, intelligence of the patient, preoperative correct diagnosis, type of operation, and mobility of the lateral pharyngeal wall. These data are supported and confirmed by our own experiences.

Most authors [22–24] agree that the *results are better in younger than in older children* or adults. Stoll et al. [25] emphasize that the best results may be attained—independent on the surgical method—if the patient is operated on or before the age of 6 years. Our opinion is that the ideal age for velopharyngoplasty is around 5 years of age: the failure of speech therapy is revealed by this age, the child is sufficiently co-operative in the days following operation which is not always easy; moreover, if chosen, postoperative speech therapy can also be commenced before the age of schooling. This effort and proposal is reflected also by our surgical statistics: most of the children operated on by us because of VPI were in the age group of 4–6.11 years. Also, the number of complications is smaller in young children than in adults [26, 27]. Of course, the choice of the time of surgery may be influenced by several factors: time of correct and final diagnosis, general health of the child, attitude and decision of the parents. Early diagnosis is essential: our aim is, therefore, to detect supposed hypernasality due to (occult) submucous cleft palate or latent VPI with the aid of nasometry of the infant cry [28]. Not evident cases can easily be overlooked by routine inspection, especially in infancy with severe consequences later on, for example, repeated adenoidectomy erroneously performed.

The *intelligence level* of the child has an unequivocal effect on the tendency of the result [22, 29]. In our opinion, the minimum IQ score should be over 50 [30, 31].

*Hearing impairment* before the operation may also affect the possible functional result; major perceptive hypacusis reduces the chances of improving speech.

**Figure 1:**
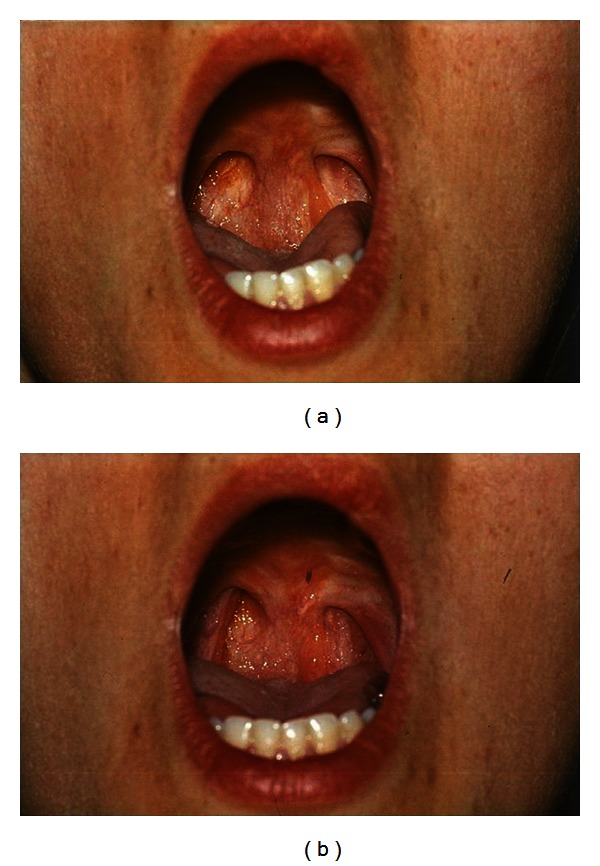
Inferiorly based pharyngeal flap in rest (a) and during sound production (b). The lateral pharyngeal walls narrow and then close the apertures beside the flap.

 To achieve a satisfactory effect with the vertical flaps, the *lateral pharyngeal wall must have a good movement *(Figures 1(a) and 1(b)).

For receiving good surgical and functional results, *proper choice of an adequate operative method is essential.*

**Figure 2:**
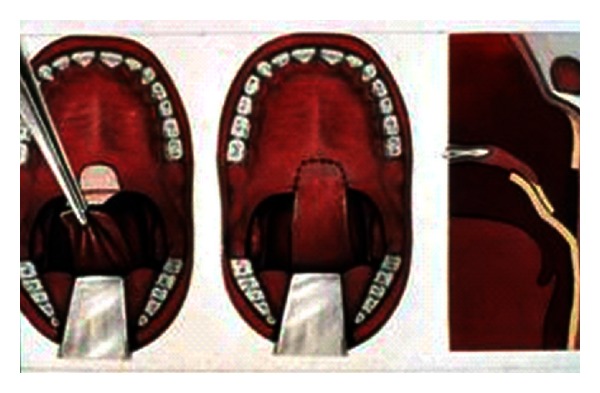
Inferiorly based pharyngeal flap.

**Figure 3:**
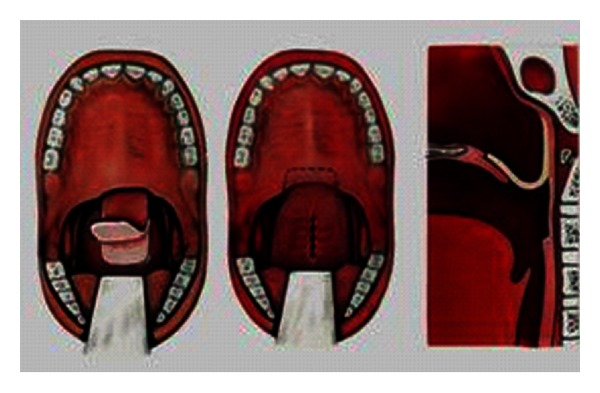
Superiorly based pharyngeal flap.

**Figure 4:**
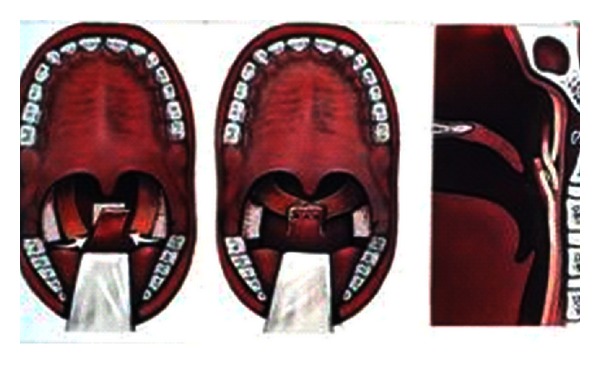
Orticochea method (sphincteroplasty).

**Figure 5:**
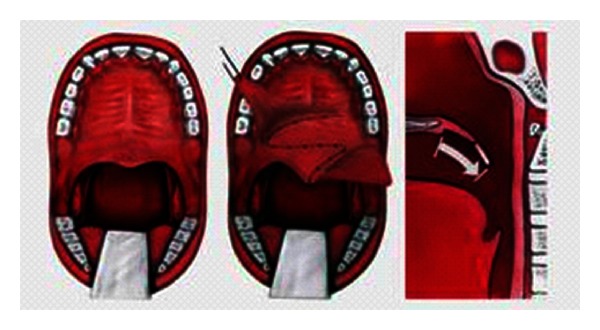
Furlow technique.

**Figure 6:**
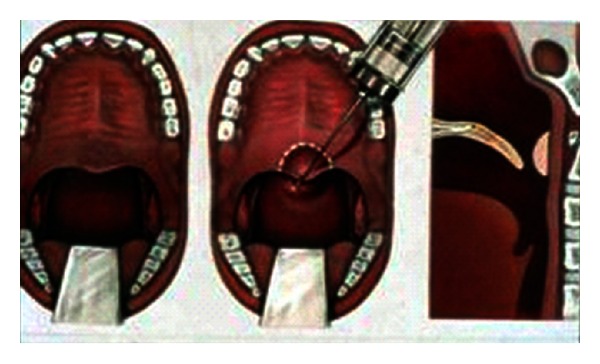
Augmentation.

**Figure 7:**
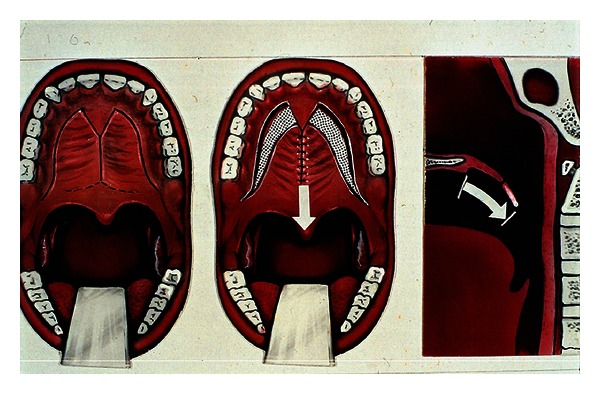
Push-back procedure.

Many great different kinds of surgical methods and techniques were described and used over the past 100 years for the surgical improvement of hyperrhinophony due to VPI (Figures 2–7):

- push-back procedures,
- pharyngoplasties which do not touch the velum,
- sphincteroplasty sec Orticochea,
- velopharyngoplasties (flap surgery),
- levator-plasty,
- Z-palatoplasty sec Furlow,
- augmentation techniques,
- modifications and combinations of the techniques.

In Figures 2–7, various surgical methods for VPI (printing with permission of Median Verlag) are shown. Undoubtedly, the velopharyngoplasties constitute the basis of palatopharyngeal operations for VPI: the so-called flap surgery is the most common among surgeries correcting hypernasality. The insertion of the pharyngeal flap into the velum can be done by different techniques; the two basic and most frequent methods are the inferiorly and the superiorly based version (Figures 2-3). Using the inferiorly based version, we lengthen the palate with an apron flap suggested by Herfert [32]. The question of which techniques is better and more successful cannot be unequivocally decided. It is a fact that more surgeons use the superiorly based method today [18, 33, 34], although some authors did achieve better functional results with the inferiorly based version [35]. According to our evaluation, the speech results are the same in both forms [6], each type has, however, some advantages and disadvantages (Table 4).

The so-called sphincteroplasty (Figure 4) is associated with the name of Orticochea [48, 49], as he himself said: “the sphincter has been discovered.” Orticochea made use of the palatopharyngeus muscles and sutured them in an inferiorly based pharyngeal flap, thus establishing the basis for sphincter-pharyngoplasty. This method became more and more popular in the last two decades and is particularly indicated when there is good movement of the soft palate, with poor movement of the lateral pharyngeal wall [50]. We obtained good results applying the Orticochea technique as a secondary operation after unsuccessful velopharyngoplasty: we attached the posterior tonsillar pillars to the rest of inferiorly based pharyngeal flap [1, 2].

The surgical technique published by Furlow [51] is an intravelar palatoplasty (Figure 5). The so-called Z-plasty means using two flaps of the velum lying opposite each other, one from the oral, the other one from the nasal layer. Thus, a muscular ring will be formed which lengthens the soft palate without using the tissues of the hard palate. This technique is suggested if the lateral pharyngeal wall functions well and the velopharyngeal gap is not larger than 7 mm.

In case of insufficiency, the VP port can be narrowed by the forward bulging (augmentation) of the posterior pharyngeal wall, too (Figure 6). Different kinds of material can be implanted at the level of the second cervical vertebra, between the submucosa and the superior constrictor muscle: Silastic, Teflon, Proplast, and biological tissues as fascia, fat, cartilage. We first used Teflon, in the last ten years autologous fat. Augmentation may be indicated:

- primarily, into the posterior pharyngeal wall, if the VP gap is less than 7 mm during phonation and the soft palate is mobil,
- secondarily, after unsatifactory result of a velopharyngoplasty. The correct localisation of implantation should be decided in these cases with the aid of oral inspection and nasopharyngoscopy.

The purpose of the push-back procedures (Figure 7) is to lengthen the secondary palate by means of mobilisation, that is, the caudal placing of the soft palate. As a result, the VP gap becomes smaller and improvement in speech is effected. Suitable cases might include those with a short palate, with adequate pharyngeal wall movement and a relatively small defect. Because of the last circumstances and restriction and because of the possible risk of maxillary growth impairment due to denudation of large areas of bone, this method is used today only in a limited number of application.

The pharyngoplasties [52], and the so-called levator plasty [53] may be used in cases of slight VPI.

In summarizing the different velopharyngeal operative methods, it may be said that a tailored, superiorly based pharyngeal flap is suggested first of all today. However, it is also correct to say that the best method is the one where a skilled surgeon has the best experience and had already achieved the best results.

The *complication rate* and the undesirable sequelae are not frequent. It must be always considered, however, that it is not about a life saving but about a speech correcting operation.

Surgical complications happen mostly at flap surgeries which need larger incision and mobilisation. Consequences of the augmentation technique are minimal [54]: infection, hematoma, throwing out, or migration of the implant may be counted to the latter. We have not experienced any side effects applying augmentation.

 Complications of pharyngeal flap surgery are also rare [55], other authors [43] stress, nevertheless, that this technique is one of the more dangerous pediatric procedures due to the potential for airway obstruction and patient death. Although airway compromise in patients who undergo pharyngeal flap palatoplasty can be a potentially fatal complication [37], I agree with Sullivan et al. [46] that this type of operation is highly successful with about 80–90% functional result and a low risk of complication. The complications are connected with the chosen anesthesia or with the surgery itself and can be classified as mild, transitory, or permanent and serious side effects.

There are some unpleasant but not serious problems postoperatively which occur in almost every patients; they are, however, *transitory* and disappear in a few days: significant pain and restriction of movement of the neck, and low fever.

Nasal obstruction, snoring, and rhinophonia clausa are also regular after surgery, but these problems gradually decrease and mostly stop as a result of the transversal contraction of the flap in some weeks or 2-3 months. Bronsted et al. [27] found hyponasality in 39% of 140 cases postoperatively, but 5 years later persistent hyponasality was revealed in only 9 patients.

*Serious (or late) complications may be the following:*

- complications in connection with the anesthesia,
- infection,
- hemorrhage,
- aspiration, tracheotomy,
- death,
- detachment of the flap,
- prolonged nasal obstruction,
- obstructive OSAS,
- disorders in maxillofacial development,
- others, rare.

The two most serious complications are hemorrhage and significant airway obstruction [6, 36].

The usual reason for major *bleeding *is that the arteria pharyngea ascendens gets injured when a wide inferiorly based pharyngeal flap is cut out, but the bleeding can generally be stopped. The flap surgery requires peculiar attention in patients with VCFS, as in these cases the carotid arteries may be medialized [56]. More serious problems are caused by the mostly diffuse bleeding in the postoperative period because it can result in the loss of the flap and become the source of grave conditions: transfusion, aspiration, pneumonia, tracheotomy may be the consequences. The number of serious hemorrhages is fortunately not too high: we observed postoperative bleeding that necessitated blood transfusion in 5 of our 1104 patients, four patients required takedown of the pharyngeal flap and we were compelled to tracheotomy in 2 cases because of significant blood aspiration. According to the literature, postoperative bleeding which claims reintubation, surgical revision or transfusion is about 0.5–2% [27, 33, 57].

*Detachment of the flap *may also occur in patients without postoperative bleeding. Insufficiency of the sutures manifests itself generally on the 8–10th postoperative days. Its cause cannot be always diagnosed. Undoubtedly, faulty surgical result of flap surgery is more frequent in patients with a very large VP gap, if the velum is cicatrized in operated CP patients, and it happens more often in adults than in young children presumably because of worse blood supply. Reoperation is possible but not with the same method; for secondary surgery, we use first of all the Orticochea technic or augmentation. We have observed that after a faulty flap surgery the speech does not worsen in every case, sometime it even improves. Explanation for this may be that the rest of the pharyngeal flap and—in case of inferiorly based version—the velum lengthened with the apron flap narrow the VP closure in some degree.

A serious *airway obstruction* with danger for suffocation can happen in the early postoperative period by aspiration of blood or—especially in retrognathic children—by retropositioning of the tongue [37, 58]. According to Willging [18], the superiorly based pharyngeal flap minimize the potential for obstructive complications associated with pharyngeal flap.

*Death* is extremely rare but cannot be excluded. The death, however, may have also general cause, it is not always in direct connection with the surgery itself [37, 59]. We have had no mortality among our 1104 operated patients.

Several authors give account of *postoperative sleep apnea (OSAS)* which is a major complication of pharyngeal flap surgery [41]. Ysunza et al. [60] found 15 OSAS with polysomnography in 585 cases after (velo-) pharyngoplasty, Valnicek et al. [36] 9 cases among 219 children. In our 1104 patient collective, we found 5 cases with postoperative OSAS verified by polysomnography. If the nasal or airway obstruction is permanent and significant and in patients with OSAS, the widening of the lateral apertures beside the flap may solve the problem. We performed this type of correction in altogether 11 cases with success, with improvement of the breathing. If this intervention is not enough, very rarely, cutting through the flap may come up. Preoperative sleep study with complete nasal obstruction with nasal tampons could be useful for predicting the risk of upper airway obstruction secondary to pharyngeal flap surgery [41]. We suggest polysomnographic investigations before pharyngeal flap surgery and also before palatoplasty in retrognathic children to predict the probable postoperative breathing problems. According to Chegar et al. [42], complications related to obstructive sleep apnea have been significantly reduced while maintaining excellent speech results by a staged approach of removing tonsils and adenoids and by creating a short, wide, superiorly based pharyngeal flap. In our practice, we routinely perform adenoidectomy before velopharyngoplasty and remove also the big tonsils for easy cutting out of the inferiorly based pharyngeal flap. In the latter case, in selected patients, some part of the tonsillar bed may be used for receptive bed of the flap during surgery.

The surgical narrowing of the VP port may *influence the maxillofacial development*, this does not result, however, in a significant deformation. It was demonstrated by us on the basis of cephalometric investigation in 53 patients [10]. The maxilla and the mandible become more retrognathic in the sagittal relation and the vertical proportion of the face increases after velopharyngeal surgery. The mandible takes up a position more down- and backward. It may be said that although pharyngeal flap surgery increases the existing sagittal and vertical distortions in UCLP and VPI patients, these do not reach a degree that would justify the abandonment of the good functional effect of veloparyngoplasty. Long and McNamara [61] also demonstrate several areas of change in facial growth following flap surgery, but this should not and does not negate its value as a desirable rehabilitative procedure. According to our study, the maxillofacial development is more propitious if operation is performed before the age of 7.

 Velopharyngoplasty produces no adverse effect on the condition or function of the ear [62, 63]; sometimes the number of serous otitis could be higher after surgery. In patients with severe perceptive hearing loss, good functional results cannot be expected, thus, in these cases surgery should not be indicated.

Table 5 summarizes the complication's rate in the material of several authors. 

In order *to prevent or at least decrease the number of complications*, the following steps are important:

- thorough preoperative examination, early and sure diagnosis,
- consideration of contraindications, and the medialized carotid arteries in cases of VCFS,
- adequate narcosis,
- correct operative technique,
- appropriate experience, practice and skill of the surgeon,
- appropriate postoperative care,
- suitable diet and supervision.

## 5. Summary

Velopharyngeal insufficiency touches many various functions, principally: deteriorates the speech. Among the speech disorders, hypernasality is the leading symptom. If this cannot be improved by speech therapy, surgery is indicated. There are several operative methods, the pharyngeal flap surgery is among them the most common procedure, a valuable tool. 1104 velopharyngeal surgeries for VPI were performed by the author during 1965–2002. The anatomical results were good: the flap built in and survived long lasting in 98.1% of the cases. In 94.1% the adhering was complete, in 4% the healing was partial with dehiscences, in 1.9% the flap detached. Regarding the functional results, hyperrinophony disappeared or became minimal in 90%, moderate in 10%; speech intelligibility proved to be excellent or good in 74%, acceptable in 21%, and poor in 5%. Articulation was excellent in 42% after surgery; the rest of the patients (1–6 sounds defective) needed further speech therapy. A tailor-made superiorly based pharyngeal flap is suggested first of all today, possibly in the age of 5 years. Although the complication rate is rare, it must be always considered that this is not a life-saving but a speech-correcting operation. The two most frequent complications are postoperative bleeding and airway obstruction. We observed among 1104 surgeries serious hemorrhage in 5 cases, aspiration in 2 cases, transfusion was necessary in 5 cases, tracheotomy in 2 cases; detachment of the flap occurred in 21 patients. Long-lasting nasal obstruction could be observed in 68 patients and OSAS in 5 cases. We had no mortality. The flap did not exert significant effect on the maxillofacial growth. In order to decrease the number of complications, careful preoperative examination, proper experience of the surgeon, adequate narcosis, well-chosen operative technique, and appropriate postoperative care are essential.

## Figures and Tables

**Table 1:** Diagnosis in 1104 cases of surgeries for VPI.

Overt cleft palate	702	63.6%
Submucous cleft palate	136	12.30
Shortening of the palate, VCFS	121	10.96
Deep nasopharynx, anatomical disproportion	54	4.90
Occult submucous cleft	3	0.27
Velar hypoplasia	6	0.54
Paresis	79	7.16
Destruction	3	0.27

**Table 2:** Distribution of 1104 surgeries according to age.

<4 years	53
4–6.11 years	641
7–9.11 years	194
10–13.11 years	134
14–18 years	60
>18 years	22

**Table 3:** Distribution of 1104 surgeries according to the used technique.

Inferiorly based pharyngeal flap	916
Superiorly based flap	148
Sphincteroplasty sec Orticochea	9
Augmentation (implantation with Teflon, later with autologous fat)	20
Furlow plasty	11
Altogether	1104

**Table 4:** Advantages and disadvantages of the superiorly and inferiorly based pharyngeal flap.

	Advantages	Disadvantages
Superiorly based flap	more physiological	more nasal obstruction
	postoperative bleeding may be stopped easier	later correction is more difficult
	larger VP gap can be bridged	

Inferiorly based flap	later correction is easier	difficult to stop postoperative hemorrhage
	less nasal obstruction	less physiological
	function well visible during speech therapy	

**Table 5:** Complications of surgery for velopharyngeal insufficiency in several publications.

Author(s), publication time	Number of patients	Diagnosis	Mean age	Type of operation	Complications
Valniček et al. 1994 [36]	219 (1985–1992)	VPI	9.6 years	Sup.based flap	bleeding: 18, reintub: 3, airway obstruction: 20, OSAS: 9, death: 1

Pena et al. 2000 [37]	88 (1983–1997)	VPI		Flap palatoplasty	airway obstruction: 7, OSAS: 1, apnea: 1, death: 1, aspiration: 2

Sie et al. 2001 [38]	48	CP, VPI syndromes	6.5 years	Furlow	palatal fistula: 2

Hofer et al. 2002 [39]	275 (10 years)			Sup. and inf. based flap	bleeding: 2, reintub.: 1, dehiscence of flap: 9

Nakamura et al. 2003 [40]	15	CP, VPI		Intravelar veloplasty	partial flap necrosis: 2

Morita et al. 2004 [41]	18	CP, VPI	children	Sup.bas.flap	OSAS: 2, tracheot.:1

Chegar et al. 2007 [42]	54 (1996–2003)	VPI	children	Sup.bas.flap	bleeding: 3, transf: 1, snoring: 4, OSAS: 0

Cole et al. 2008 [43]	222	VPI	6.4 years	Sup.phar. flap	infection: 1, bleeding: 3, OSAS: 5

Keuning et al. 2009 [33]	130	VPI		Sup.bas.flap	bleeding: 1, dehiscences: 3

Ysunza et al. 2009 [44]	29 (2000–2007)	VCFS		Flap: 20, Sphincter: 9	no complications

Leuchter et al. 2010 [45]	18 (2004–2007)	mild VPI		Augmentation, diff. implants	hematoma: 1, cervical pain: 1

Sullivan et al. 2010 [46]	104 (1981–2008)	CP, VPI	8.6 years	Sup.bas.flap	OSAS: 2

Kilpatrick et al. 2010 [47]	36 (2003–2009)	CP,VCFS	8.1 years	Sphincteroplasty	fever: 2, bleeding: 1, allergy: 1, O_2_ therapy: 4
